# American Indian and Alaska Native violence prevention efforts: a systematic review, 1980 to 2018

**DOI:** 10.1186/s40621-024-00488-3

**Published:** 2024-03-19

**Authors:** Jeffrey E. Rollman, M. Thomas, Laura M. Mercer Kollar, Katie A. Ports, Carmen Clelland, Delight E. Satter, Corinne David-Ferdon

**Affiliations:** 1 Fielding School of Public Health, University of California Los Angeles, Los Angeles, CA 90095 USA; 2 Independent Researcher, Gulf Breeze, FL 32563 USA; 3 Division of Violence Prevention, Centers for Disease Control and Prevention, National Center for Injury Control and Prevention, 4770 Buford Highway, Atlanta, GA 30341 USA; 4 American Institutes for Research, 10 South Riverside Plaza, 6th Floor, Chicago, IL 60606 USA; 5 Indian Health Service, 5600 Fishers Lane, Rockville, MD 20857 USA; 6 Centers for Disease Control and Prevention, National Center for State, Tribal, Local, and Territorial Public Health Infrastructure and Workforce, 1825 Century Boulevard, Atlanta, GA 30345 USA; 7 Centers for Disease Control and Prevention, National Center for Injury Prevention and Control, 4770 Buford Highway, Atlanta, GA 30341 USA

**Keywords:** American Indian or Alaska Native, Violence, Violence prevention, Systematic review, Suicide

## Abstract

**Background:**

Violence is a serious public health concern disproportionately experienced by American Indian and Alaska Native (AIAN) people. While the burden and impact of violence may be explained by the presence of risk factors among this group, AIAN communities benefit from unique protective factors and universal strategies which may be tailored with tribal adaptations. We sought to identify and explore violence prevention strategies specific to AIAN populations.

**Methods:**

A review was conducted to systematically identify violence prevention programs, policies, and practices implemented in AIAN communities. We searched nine electronic databases and relevant gray literature released between January 1980 and June 2018. We included intervention-focused records targeting at least one violence topic area (child abuse/neglect, elder abuse, intimate partner violence, sexual violence, youth violence, and suicide) in a majority (> 50%) AIAN population.

**Results:**

A total of 5220 non-duplicate records were screened, yielding 318 full-text records. After applying exclusion criteria, 57 records describing 60 program, policy, or practice implementations of 43 unique interventions were identified. All six violence types were represented, although more than half (58%; *n *= 25/43) focused on suicide prevention. Among suicide prevention programs, the most common strategies were identifying and supporting people at risk (80%; *n *= 20), teaching coping and problem-solving skills (56%; *n *= 14), and promoting connectedness (48%; *n *= 12). Two-thirds of the implementations (67%; *n *= 40/60) were in fully (100%) AIAN communities. Programs were implemented across many settings, though schools were the most common (35%, *n *= 21/60) setting. Of the 60 total implementations, a majority (80%; *n *= 48) were new approaches developed by and for AIAN communities, while the remainder were AIAN adaptations of programs previously created for non-AIAN populations. Most implementations (60%; *n *= 36/60) provided some evaluation data although less than half (45%; *n *= 27/60) reported evaluation results.

**Conclusions:**

This review identified many violence prevention strategies specific to AIAN populations. While programs developed in one tribe may not be completely generalizable to others, shared tribal risk and protective factors suggest programs could be successful across diverse communities. Findings indicate there is a need to develop and evaluate violence prevention programs, policies and practices for AIAN populations.

**Supplementary Information:**

The online version contains supplementary material available at 10.1186/s40621-024-00488-3.

## Background

Violence in its many forms (e.g., child abuse and neglect, youth violence, intimate partner violence, sexual violence, elder abuse, and suicide) is a serious public health issue. In 2020, over 71,000 people in the USA died as a result of violence, while approximately 2.0 million people were treated in emergency departments for violence-related injury (CDC 2021). Exposure to violence has lasting effects on the physical and mental health of individuals, including depression and anxiety, substance use, chronic and infectious diseases, and life opportunities, such as educational attainment and employment (Shonkoff et al. 2012; Metzler et al. 2017; Hillis et al. 2017). Community factors (e.g., poverty, limited access to high-quality education, unstable housing) can contribute to increased risk for violence and other health problems and the differences observed across groups (US Department of Health and Human Services 2020). American Indian and Alaska Native (AIAN) people experience disproportionate rates of violence (Leavitt et al. 2018; Petrosky et al. 2020, 2021; Kegler et al. 2022). AIAN communities are also more likely to experience adverse childhood experiences, potentially violent and traumatic events that occurs in childhood which may include experiencing violence, child abuse, or neglect; witnessing violence in the home or community; economic and rural hardship; and income deprivation, which increase the risk of experiencing violence and other health problems throughout the lifespan (Anda et al. 1998; Miller et al. 2011; Ports et al. 2016; US Department of Health and Human Services 2015). In 2020, suicide was the leading cause of death in AIAN aged 10–14 and the second leading cause of death for AIAN aged 15–34 years; homicide was the eighth leading cause of death for AIAN females and sixth leading cause of death for AIAN males aged 1–54 years (CDC 2021).

Different forms of violence (e.g., child abuse and neglect, youth violence, intimate partner violence, sexual violence, elder abuse, and suicide) share similar risk and protective factors that accumulate throughout childhood, adolescence and adulthood (Wilkins et al. 2014). Additional risk factors, including contemporary reminders of historical trauma experienced by AIAN populations (e.g., loss of land, language, traditions and respect for traditional ways), contribute to inequities in exposure to risk factors for violence (Evans-Campbell 2008; Bombay et al. 2013; Mohatt et al. 2014; Whitbeck et al. 2004). Despite noted inequities, AIAN communities remain resilient and possess cultural and community assets that can be protective against violence, including community-mindedness, connection to tribal leaders, tribal language, participation in tribal ceremonies, and spirituality (Henson et al. 2017; Wright et al. 2013). The burden and impact of violence on AIAN communities, as well as their unique risk and protective factors, highlight the need for culturally specific violence prevention strategies. Culturally specific frameworks for violence prevention have been developed (Whitbeck et al. 2004; Dapice 2006; Robin et al. 1996), but effectively identifying and implementing culturally specific prevention strategies across varying forms of violence is a gap in the public health approach to violence prevention (Antone et al. 2016; Satter et al. 2021; Solomon et al. 2022).

Identifying effective violence prevention strategies, evaluating existing strategies, and developing new strategies to address gaps in the violence prevention field are critical to widespread violence prevention in AIAN communities. While prevention approaches and strategies are broadly identified in CDC’s Technical Packages for Violence Prevention (Center and for Injury Prevention and Control 2021), none were specifically described for AIAN populations. Thus, a comprehensive systematic review of violence prevention initiatives is a critical first step in identifying existing efforts and highlighting gaps specific to AIAN people and communities. Although suicide-specific systematic reviews for AIAN communities exist (Middlebrook et al. 2001; Clifford et al. 2013), there are currently no reviews which examine prevention programs across all violence topic areas. Decades of research have shown that multiple forms of violence are interconnected; therefore, adopting a shared risk and protective factors framework and reviewing violence prevention programs for one form of violence can inform the prevention of other forms of violence (Centers for Disease Control and Prevention 2016). This review was conducted to systematically identify violence prevention strategies and approaches for AIAN communities and to inform current and future prevention practices, identify literature gaps, and guide future research.

## Methods

We used PRISMA guidelines (Liberati et al. 2009) and other recommended protocols (Booth et al. 2012) to conduct a systematic review of published and unpublished records to identify violence prevention programs, policies and practices for AIAN communities. Peer-reviewed articles, dissertations, theses, and governmental and nongovernmental publications published from 1980 to 2018 were identified through a search of nine electronic databases: Medline (OVID), PsycInfo (OVID), CINAHL (EBSCO), Sociological Abstracts (ProQuest), NTIS (EBSCO), Scopus, Criminal Justice Database (ProQuest), ERIC (ProQuest) and Academic Search Complete (EBSCO). A manual review of selected relevant journals (i.e., International Journal of Indigenous Health, American Indian and Alaskan Native Mental Health Research, Washington University Journal of American Indian and Alaska Native Health, American Indian Culture and Research Journal) and articles in Google Scholar were conducted to ensure publications (2016–2018) that may not have been cataloged yet were included in the review. A search was also conducted of the Indian Health Service’s (IHS) unindexed publication, the IHS Primary Care Provider (Indian Health Service 2018), to ensure all relevant gray literature (e.g., annual and research reports, white papers) from 1980 to 2018 was included.

These sources were examined for records related to AIAN, violence, and prevention and intervention strategies (Appendix A). We included violence search terms from relevant literature (Wilkins et al. 2014; Degue et al. 2014; Holman et al. 2016). We also included a variety of search terms that captured prevention and intervention terminology identified from similar systematic reviews (Clifford et al. 2013; Degue et al. 2014; Holman et al. 2016).

**Fig. 1:**
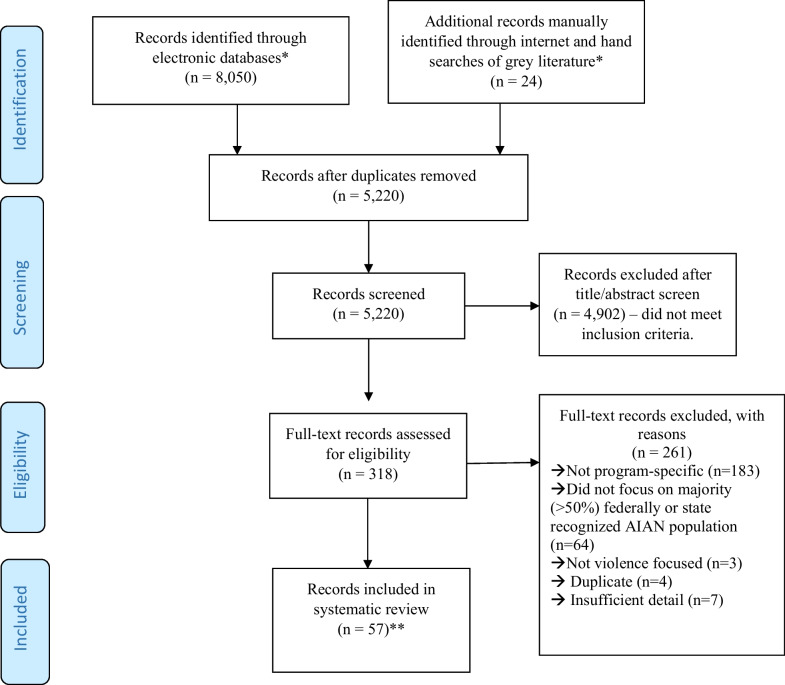
Flowchart of American Indian and Alaska Native Violence Prevention review 1980–2018. *Note Electronic databases searched include: Medline (OVID), PsycInfo (OVID), CINAHL (EBSCO), Sociological Abstracts (ProQuest), NTIS (EBSCO), Scopus, Criminal Justice Database (ProQuest), ERIC (ProQuest), ProQuest Central, Academic Search Complete EBSCO. Manually identified from hand review/search of non-indexed literature include: Google Scholar, IHS Primary Care Provider Journal and gray literature culled from ancestry and forward citing of included publications; Two year manual review of selected relevant and unindexed journals (International Journal of Indigenous Health; American Indian and Alaskan Native Mental Health Research; Washington University Journal of American Indian and Alaska Native Health). **Note The 57 records covered 60 unique implementations, as some studies reported multiple programs, policies and practices

A total of 8,074 records were identified (Fig. 1). After duplicates were removed, 5,220 abstracts were reviewed by two co-authors to determine if they met inclusion criteria. Inclusion criteria included: 1) product published between January 1980 and June 2018; 2) written in English; 3) at least one violence topic area (i.e., child abuse and neglect, elder abuse, intimate partner violence, sexual violence, suicide, youth violence) was the primary focus of a prevention or intervention strategy; and 4) the majority (> 50%) of participants were identified as members of a US federally recognized or state recognized tribe (J. T. 2018). We did not include records that focused on Native Hawaiian or Pacific Islander populations. Exclusion criteria included: 1) record did not describe the development or implementation of a program, policy, or practice; 2) prevention or intervention focused exclusively (> 75%) on unintentional injury (i.e., accidental poisoning/overdose, motor vehicle collision) or another health issue (e.g., substance use); 3) the results and discussion of the record did not focus on a majority (> 50%) federally recognized or state recognized AIAN population; 4) the record provided insufficient detail about the program, practice or policy implemented to determine if it met all inclusion criteria; and 5) record was a study review, meta-analysis, or protocol that lacked information about the prevention program. Program evaluation was not a criterion for inclusion, and not all programs listed below have been evaluated. Following the abstract review, 4,902 records were excluded.

Two research team members conducted a full-text review of 318 records to determine eligibility for inclusion with 100% overlap for consensus agreement using inclusion and exclusion criteria above. Of the 318 full-text records reviewed, 261 were excluded (e.g., record not program specific, not focus on majority AIAN population; see Fig. 1), and 57 met inclusion criteria for the systematic review (Fig. 1).

The 57 included records were coded for: (1) violence type (child abuse/neglect, youth violence, intimate partner violence, sexual violence, elder abuse and/or suicide); (2) publication type (e.g., peer-reviewed publication, government publication); (3) violence prevention strategy (included from CDC technical packages); (4) strategy or program origin (e.g., adapted from existing program or new strategy); (5) study population demographics (e.g., tribal setting location, age (years) or education year); (6) study design (e.g., program evaluation, randomized controlled trial, implementation phase, comparison group presence); (7) setting where the program was implemented (e.g., school, medical/clinical, justice system, social services, lay community led, government policy); and 8) results (e.g., outcomes of interest measured, methods for measuring outcome). Violence prevention strategies were identified using relevant technical packages based on the form(s) of violence identified (e.g., for intimate partner violence, an identified strategy would be support survivors to increase safety and lessen harms). While there is no technical package for elder abuse prevention, the study team mapped the identified interventions to the closest technical package strategy available across the technical packages (i.e., teach skills). Descriptive analyses were calculated via percentages.

## Results

This systematic review identified 60 program, policy, or practice implementations or approaches across 57 records as some records had more than one unique program, practice, or policy resulting in a greater number of total implementations or approaches (*n *= 60) than records (*n *= 57). Implementations is a comprehensive term to include program, policy, or practice described. Of the 60 implementations, 72% (*n *= 43/60) were unique or non-duplicative programs (e.g., American Indian Life Skills, Discovery Dating, Pathways to Hope). Specific programs, policies or practices may have appeared more than once, such as being implemented in two different communities, and thus, the program itself is only counted once as a unique, non-duplicative program (*n *= 43). Fifteen implementations (25%, *n *= 15/60) involved the same program in different settings and/or with different groups (e.g., American Indian Life Skills was implemented or evaluated in three records; see Additional File 1).

### Publication type

Peer-reviewed journal articles accounted for 67% (*n *= 40/60) of total implementations. Government publications made up 13% of the included records (*n *= 8/60), with all but one identified through the IHS Primary Care Provider. Theses and dissertations made up 7% (*n *= 4/60). Gray literature made up 13% (*n *= 8/60). For certain violence types, government publications and dissertations were also sources of program information. For example, 20% (*n *= 4/20) of the 20 programs, policies or practices focused on sexual violence or intimate partner violence were reported in government publications and 5% (*n *= 1/20) in theses or dissertations.

Settings were also identified in different publications. While peer-reviewed journal articles accounted for most of the 21 reported programs in schools (67%, *n *= 14/21) and 17 reported programs in community organizations (82%, *n *= 14/17), other publication types were important when gathering information about clinic-based programs and programs implemented in other settings. For instance, government publications (*n *= 8) provided information about three programs (60%, *n *= 3/8) implemented in outpatient clinics. Information about programs, policies or practices in other settings (*n *= 14) came equally from peer-reviewed journal articles (*n *= 7/14) and other publications (*n *= 7/14), with government publications (21%, *n *= 3/14), gray literature (21%, *n *= 3/14), and dissertation/thesis (7%, *n *= 1/14) accounting for the remaining records.

Ten (17%, *n *= 10/60) implementations were focused on youth under 18 years of age. The vast majority or 78% (*n *= 47/60) of programs, policies or practices were newly developed approaches, 20% (*n *= 12/60) were adapted from previously developed approaches, and 2% (*n *= 1/60) did not identify approach origin. Twenty-five percent (*n *= 15/60) of approaches used a community-based participatory research model, and 60% (*n *= 36/60) reported evaluation results.

### *Unique programs, policies, or practices (n *= *43)*

#### Violence category

Six violence types were represented among the 43 unique or non-duplicative programs, policies or practices. More than half (*n *= 25; 58%) of unique programs, policies or practices focused on suicide prevention (see Additional File 1). The other prevention programs, policies or practices focused on child abuse and neglect (*n *= 3; 7%), youth violence (*n *= 4; 9%), intimate partner and/or sexual violence prevention (*n *= 4; 9%), and elder abuse (*n *= 1; 2%). Six (14%) programs, policies or practices identified focused on the prevention of more than one form of violence. For instance, an evaluation of policies to reduce excessive use of alcohol in Alaska Native villages examined impact on rates of both suicide and interpersonal violence (Wood and Gruenewald 2006).

#### Violence prevention strategy

Many of the identified programs, policies or practices align with multiple violence prevention strategies (e.g., a program focused on suicide prevention may promote connectedness and teach coping and problem-solving skills). Of the 25 suicide prevention programs, policies, or practices, the most common strategies were identifying and supporting people at risk (80%; *n *= 20), promoting connectedness (48%; *n *= 12), and teaching coping and problem-solving skills (56%; *n *= 14). By contrast, only five (20%) created protective environments (e.g., reducing access to lethal means among persons at risk for suicide). Only one program (4%) focused on improving access to and quality of suicide care.

Of the three programs, policies or practices focused on preventing child abuse and neglect, two align with the strategy to enhance parenting skills to promote healthy child development and two aligned with the strategy to intervene to lessen harms and prevent future risk. Youth violence prevention programs, policies or practices (*n *= 4) involved strategies to connect youth to caring adults and activities (*n *= 2) and strengthen youth’s skills (*n *= 2). Programs, policies or practices focused on preventing intimate partner/sexual violence (*n *= 4) included strategies to teach safe and healthy relationship skills (*n *= 2), teach skills to prevent sexual violence (*n *= 2), and engage influential adults and peers (*n *= 2). The only program focused on elder abuse utilized the strategy of teaching skills to families and caregivers to prevent elder abuse. Crosscutting programs, policies or practices (*n *= 6) included strategies to strengthen youth’s skills generally (*n *= 3) and specifically teaching coping and problem-solving skills (*n *= 4).

### *Total program, policy, or practice implementations (N* = *60)*

Recall some records had more than one unique program, practice, or policy resulting in a greater number of total implementations or approaches (*n *= 60) than records (*n *= 57). This section pertains to the overall number of implementations (*n *= 60).

### American Indian/Alaska Native participant demographics

There were 14 named tribes among the AIAN communities where 60 records of program, policy, or practice creation, evaluation, or implementation took place. Many described programs, policies or practices (*n *= 25) that did not specifically identify a tribe in the publication. Programs, policies or practices were implemented in more than 14 states, the majority of which are in the western region of the USA. Four implementations (7%) did not provide specific state or tribal information but did provide broad geographic information (e.g., Midwestern, Western, High Plains and Northern Plains regions).

Two-thirds of the programs, policies or practices (*n *= 40; 67%) were implemented in populations composed fully (100%) of American Indians and/or Alaska Native people. Another 27% (*n *= 16) did not specifically list the percentage of AIAN people in the evaluation sample, but the setting implied a majority AIAN population (e.g., conducted in a tribal community). Of the remaining four programs, policies or practices, two had evaluation populations which had majority AIAN participants (54% and 90%). The other two, though they used data sets which did not have a majority of AIAN respondents (2.7% and 40% AIAN), focused primarily on AIAN respondents when writing the results and conclusions (Bartgis and Albright 2016; Chaffin et al. 2012). Thirteen of the 60 programs, policies or practices (22%) reported the mean age of their study population, which ranged from 11.1 to 48.1 years.

### Reported settings involved in program, policy, or practice implementation

The most common location of programs, policies or practices reported taking place in schools (35%, *n *= 21; note not every record reported age). Five (8%) were implemented in outpatient clinics. One program was implemented through changes to law enforcement and one in a social services agency. One program did not provide sufficient information to determine the setting.

There were no programs, policies or practices centered exclusively in inpatient hospitals; however, that setting was included as a part of some programs, policies or practices. For instance, one suicide prevention program adaptation included an emergency-department intervention as part of continued psychological care and home visits for people who attempted suicide (Cwik et al. 2016a). There were no programs, policies or practices focused exclusively on changing government policies, though one study described the role of local alcohol policies, among other activities, in preventing violence (Wood and Gruenewald 2006). There were no programs, policies or practices directly reported to be led by tribal elders; however, 17 programs (28%) were reported to take place in community centers or gathering spaces and to be led by community members, of which tribal elders may well have been a part. For example, tribal elders were intimately involved in the development of the Qungasvik Yup’ik Intervention Toolbox, though the resulting activities were led by unspecified tribal community member facilitators (Rasmus et al. 2014). Fourteen programs (23%) involved multiple settings (e.g., a suicide-risk surveillance system, which includes reports from schools, emergency departments, and individuals).

### Program, policy, or practice development

A majority (80%, *n *= 48) were new approaches developed by and for tribal communities. Some of these, such as Zuni Life Skills and Pathways to Hope, were later adapted by and implemented in other tribes (Buhs 2000; LaFromboise and Fatemi 2011; Payne et al. 2013). Eleven programs (18%) were implementations or adaptations of programs previously created for non-AIAN audiences. Of these, seven were originally developed for non-AIAN audiences and did not significantly adapt the program curriculum for the AIAN audience. For instance, SafeCare included brief training of providers about American Indian culture, but no adaptations were made to the SafeCare model or its curriculum materials (Chaffin et al. 2012). The remaining four programs made significant adaptations to curricula to increase relevance for the AIAN community, with three using community-based participatory research in the adaptation (Cwik et al. 2016a; Goodkind et al. 2012; Richmond et al. 2008). These programs included an adaptation of the emergency-department-based intervention for adolescents who attempt suicide, which focused largely on accounting for the increased burden on and limited capacity of emergency departments serving AIAN communities (Cwik et al. 2016a). With the adaptation of the group intervention Cognitive Behavioral Intervention for Trauma in Schools, the program emphasized incorporating historical trauma and cultural protective factors into the curriculum (Goodkind et al. 2012). An adaptation of Safe Dates included curriculum changes to ensure cultural relevance and development of culturally specific evaluation measures (Richmond et al. 2008). The final adaptation, which implemented a youth violence prevention curriculum called Families and Schools Together, adapted the curriculum to include cultural protective factors, such as traditional songs, dance, food, and connection to community elders, in tribal schools (Kratochwill et al. 2004). One program did not provide sufficient information to determine whether it was an adaptation or a novel program (Alaska: Program 2016).

### Program, policy, or practice evaluation data and methodology

A total of 36 implementations reported some evaluation data, 27 reported complete evaluation data, and nine provided limited or incomplete evaluation data. An example of limited or incomplete data includes a statement of “significantly positive outcomes…on measures of life skills efficacy, depression management, stress management, ability to enlist community support, and ability to enlist social resources” (LaFromboise and Malik 2016) without any numerical or statistical information or “rapes reduced by 30%” (LaFromboise and Malik 2016) without further context or methodology (Alaska: Program 2016). Of the programs, policies or practices which did include some evaluation data, 27/36 (75%) were peer-reviewed journal articles. The other program, policy, or practice evaluations appeared in government publications (*n *= 3/36; 8%), dissertations or theses (*n *= 2/36; 6%), and gray literature (*n *= 4/36; 11%).

Of the 36 implementations reporting at least some evaluation data, 78% (*n *= 28) reported the methods used to collect and analyze data, and 22% (*n *= 8) used randomized control trials with a comparison control condition when evaluating impact. Thirteen (30%) of the 43 unique programs, policies or practices included evaluation data. Of these, two had comprehensive evaluation data collected beyond an immediate post-evaluation. The American Indian Life Skills adaptation of the Zuni Life Skills suicide prevention program was described in five publications, three of which included evaluation data finding significant reductions in suicide risk and suicidal ideation (LaFromboise and Fatemi 2011; LaFromboise and Howard-Pitney 1995).

## Conclusions

This systematic review of 38 years of AIAN violence prevention records identified 57 records reporting 60 programs, policies or practices and of those, 43 were unique (non-duplicative) programs, policies or practices. This review revealed gaps where more work is needed to identify AIAN violence prevention promising practices across violence types and to conduct evaluation for effectiveness. Below, a discussion of strengths, challenges, limitations and future recommendations is provided.

### Strengths of AIAN violence prevention efforts

This review identified 43 unique programs, policies and practices that addressed violence prevention for AIAN persons. They addressed different forms of violence and used a range of prevention strategies identified in CDC’s technical packages for violence prevention (Center and for Injury Prevention and Control 2021). Additionally, programs, practices and policies were both novel and adapted, suggesting there are different ways that culturally specific violence prevention efforts may be implemented in AIAN communities.

Programs, practices and policies included implementations in communities and schools to reach youth and families, with some programs focusing on future generations via youth violence (*n *= 4) and child abuse and neglect prevention efforts (*n *= 3; see Additional File 1). These programs incorporated a variety of violence prevention strategies, ranging from strengthening parenting or youth’s skills to promoting early interventions to prevent harms. Ensuring the healthy development of youth can reduce the risk for future violence and other associated health problems (Centers for Disease Control and Prevention 2016). Implementations were geographically diverse and different tribes were represented, suggesting this work occurs across tribal communities. Some program, policy, and practice evaluation data were reported, some mentioned an evaluation and had no data, and others had no evaluation data available.

### Limitations

#### Review limitations

Findings are limited to the time frame of 1980–2018 as this review was part of conference proceedings and does not include an update for more recent years. Limitations also included that the examined published work may be an undercount of actual violence prevention programs available in AIAN communities. This may be related to a number of factors, including unpublished impacts of implemented programs, community driven programs that did not result in publication of findings, and complicated norms and jurisdictional issues (e.g., data sovereignty, protecting tribal knowledge). Additionally, this review focused on violence prevention and did not include prevention efforts that primarily addressed risk and protective factors (e.g., academic achievement, substance use) that may also have a primary prevention effect on violence.

#### Program, practice, and policy limitations

There were several limitations in the identified publications that help point to where continued and strengthened program development, implementation, and evaluation can occur. For instance, when this review was conducted, there were 573 federally recognized and 63 state recognized tribes. Many program, policy, or practice implementations or approaches occurred or were each evaluated within a single tribe; thus, results may not be generalizable to other tribes. Not all implementations were evaluated, and when evaluation evidence was available often the study design had methodological challenges (e.g., no control or comparison groups, small study populations, small convenience samples). More work is needed to further understand evaluation challenges.

### Challenges and future opportunities of AIAN violence prevention efforts

While violence prevention efforts in AIAN communities are ongoing, more work remains. Few published programs were identified that were implemented to address child abuse and neglect, youth violence, intimate partner violence or sexual violence, all of which are prevalent forms of violence in AIAN communities. While several suicide prevention approaches were identified, the continued high rate of suicide attempts and related deaths among AIAN people suggests more effort is needed to design and implement effective strategies (Leavitt et al. 2018; Kegler et al. 2022; US Department of Health and Human Services 2021). CDC’s violence prevention technical packages help to identify many strategies and approaches that could be explored to fill these gaps by implementing and/or culturally adapting and evaluating identified violence prevention strategies and approaches (Center and for Injury Prevention and Control 2021). Future work may also identify effective strategies and approaches developed within AIAN communities to prevent violence and examine effectiveness.

Evidence was present that previously developed effective prevention strategies are being implemented in AIAN communities, at times with adaptation to increase potential relevance to the community’s culture and traditions and to address salient issues, such as historical trauma. Adaptation may be a cost-effective approach versus developing new programs for every unique community, and more culturally specific evaluation is needed to verify effectiveness of adapted strategies and approaches on preventing and reducing violence outcomes among AIAN persons and communities. More work is needed to determine to what extent adaptations are critical to generalize prevention effects when implemented in new communities. It is unclear which facets of program implementations can be generalized universally and which strategies necessitate culturally unique tribal specific adaptations. Further, identifying core program elements to inform adaptations, incorporating traditional and cultural practices, and evaluating violence prevention efforts are needed to address the experiences of the diverse federally recognized tribes and numerous state recognized tribes. Determining culturally responsive ways to identify promising and best practices and disseminate these programs to other communities remains a challenge and area for future work that can be informed by this review. Existing literature demonstrates the development and implementation of violence prevention programs, policies, and practices in collaboration with AIAN communities is possible (Rasmus et al. 2014; LaFromboise and Howard-Pitney 1995).

Published reports also demonstrated that the design and implementation of violence prevention programs remain complicated given structural challenges (e.g., lack of funding, institutionalized challenges) and other barriers (e.g., protection of sovereignty, protection of sacred and cultural knowledge) (Antone et al. 2016; Satter et al. 2021; Solomon et al. 2022). Rigorous evaluation of approaches is needed but is also a challenge for multiple reasons including, but not limited to, structural challenges, small populations sizes, lack of trust with Western science study methods including randomized controlled trials, and respecting tribal data sovereignty (Satter et al. 2021; Middlebrook et al. 2001). Evaluation is also hindered by funding constraints, perceived and/or actual evaluation challenges, and difficulty in identifying shared outcomes of interest. For example, standard evaluation procedures may fail to capture short-term effects that tribes may prioritize, such as community involvement, adoption of cultural customs, improved understanding of culture and history, healthier and/or more equitable policies and environments and ‘evidence-based practices’ built on time immemorial traditional knowledge and practice (Mohatt et al. 2014; Kelley et al. 2015). Building and strengthening an understanding of culturally responsive evaluation within the context of violence prevention may move forward best and promising violence prevention efforts whether developed or adapted by communities (Kratochwill et al. 2004).

This review highlighted a number of important considerations for implementing violence prevention efforts with AIAN people. Identifying cultural and traditional elements, aligning programs with future generations using the shared risk and protective factors frameworks, and building community collaborations incorporates and acknowledges the expertise of the community and its ability to develop and adapt culturally appropriate, trauma-informed, and responsive efforts. There remain many areas of violence prevention that were not represented in our review, such as strategies to strengthen family economic supports and early childhood education, so identifying key needs within communities and building violence prevention efforts are needed (Center and for Injury Prevention and Control 2021). Broadly, there is a need for increased availability of evidence-based violence prevention programs, policies and practices for all forms of violence for AIAN people and communities.

### Supplementary Information

**Additional file 1: Table.** Programs, Policies, and practices organized by violence type and publication year.

## Data Availability

The datasets used and/or analyzed during the current study are available from the corresponding author on reasonable request. All included articles may be found in Additional file 1.

## Appendix A: Medline query search terms

Exp American Native Continental Ancestry Group/ OR (Native American* OR American Indian* OR Alaska Native* OR Alaskan Native* OR Native Alaskan* OR Inuit* OR Tribal Nation* OR First Nations OR Indian reservation* OR Tribal government* OR tribal college* OR tribal universit* OR Indian Health Service).ti,ab.

AND

Violence OR assault* OR spouse abuse OR spousal abuse OR battered OR elder abuse OR child abuse OR abusive behavior* OR rape OR firearm injur* OR homicide* OR suicide* OR self-harm OR self-injur* OR harmful behavior OR maltreatment OR child neglect OR elder neglect OR juvenile delinquen* OR aggression OR aggressive behavior* OR antisocial behavior* OR violent behavior* OR fighting OR gang* OR bullying OR bullied OR bullies

AND

Prevention OR intervention* OR education OR program* OR curricul*

Note: Queries using the same search terms were conducted in other electronic databases.

## Abbreviations

AIAN: American Indian or Alaska Native
CDC: Centers for Disease Control and Prevention (USA)
EBSCO: EBSCO search engine database used by libraries
IHS: Indian Health Service (USA)
OVID: Ovid search engine database used by libraries
ProQuest: ProQuest search engine database used by libraries
