# Supplementary material for: American Indian and Alaska Native violence prevention efforts: a systematic review, 1980 to 2018
Source: Inj Epidemiol. 2024 Mar 19;8(Suppl 2):72. doi: 10.1186/s40621-024-00488-3 (PMC10949553; doi:10.1186/s40621-024-00488-3)
Supplement: Supplementary file 1 — Additional file 1: Table. Programs, Policies, and practices organized by violence type and publication year. [file 40621_2024_488_MOESM1_ESM.docx]

**Additional File 1**

**American Indian and Alaska Native Violence Prevention Efforts: A Systematic Review, 1980 to 2018**

**Table: Programs, Policies, and Practices Organized by Violence Type and Publication Year**

| ***Program, Policy, or Practice Name***  **(Reference Number)**  **First author, publication year^1^** | **Tribal Setting**  **Location**  **Age (years) or Education Year^2^**  (if available) | **CDC Prevention Strategy^3^** | **Specific Activities^4^** | **Outcome(s) Evaluated or Mentioned^4^** |
| --- | --- | --- | --- | --- |
| **Suicide (n = 25 unique implementations)** | | | | |
| *Zuni Life Skills (ZLS)* [50,52,53]  *LaFromboise et al. (1994)*  *LaFromboise et al. (1995)*  *LaFromboise (2006)* | -Zuni Pueblo  -New Mexico  -Mean Age 15.6 | -Promote connectedness  -Teach coping and problem-solving skills | -life skills training and education about youth suicide in Zuni Pueblo community  -psycho-education, coping skills enhancement, lifestyle change activities  -cognitive and emotional approach coping through positive thinking and effective problem-solving strategies | -Suicide risk factors |
| *American Indian Life Skills (AILS)* [42,48,54]  Buhs (2000)  LaFromboise (2011)  LaFromboise et al. (2016) | -Cherokee Nation; Northern Plains tribe  -New Mexico; Oklahoma; South Dakota  -Ages 14-18 | -Promote connectedness  -Teach coping and problem-solving skills | -life skills training and education about youth suicide in AIAN tribal communities  -psycho-education, coping skills enhancement, lifestyle change activities  -cognitive and emotional approach coping through positive thinking and effective problem-solving strategies | -Suicide risk factors |
| *National Model Adolescent Suicide Prevention* [55–57]  May et al. (1994)  DeBruyn et al. (1997)  May et al. (2005) | -Jicarilla Apache  -New Mexico  -Ages 11-18 | -Promote connectedness  -Teach coping and problem-solving skills  -Identify and support people at risk  -Improve access and quality of suicide care | -surveillance through constant data and information gathering  -screening/clinical interventions in health clinics, schools  -school-based life skills development and prevention programs  -peer training, referral, and counseling  -increased professional mental health provider staff | -Suicide attempts  -Self-harm behaviors |
| *American Indian/Alaska Native Community Suicide Prevention Center and Network* [56]  DeBruyn et al. (1997) | -Jicarilla Apache  -New Mexico | -Teach coping and problem-solving skills  -Identify and support people at risk  -Lessen harms and prevent future risk | -trainers from AIAN communities assist tribes in planning suicide prevention programs  -trainings on suicide crisis/grief intervention, school-based suicide prevention, crisis response, surveillance, program evaluation | -no evaluation data available |
| *Suicide Intervention Team* [56]  DeBruyn, et al. (1997) | - Midwestern tribe | -Identify and support people at risk | -train and provide continuing education opportunities for volunteer team  -respond to suicide attempts with contact during the crisis, emergency room support, 72-hour protective services, listener resources, referral to mental health services | -Suicide attempts |
| *Suicide Prevention and Intervention Program* [56,58]  DeBruyn et al. (1997)  Centers for Disease Control and Prevention MMWR (1998) | -Western Athabaskan  -New Mexico  -Age 15-19 | -Teach coping and problem-solving skills  -Identify and support people at risk | -School-based “natural helpers” team of trained students who respond to youth in crisis and notify professionals of the need for assistance  -“Natural helpers” provide peer-based education on self-esteem, team building, and suicide prevention | -Suicide attempts  -Deaths by suicide |
| *IHS National Suicide Prevention Committee Work Plan* [59]  Stuart (2005) | -National IHS program | -Promote connectedness  -Identify and support people at risk | -community education and outreach led by tribal providers (therapists, school personnel, health providers, social services, injury prevention specialists, traditional healers)  -clinical assessment of at-risk behavior and delivery of effective treatment  -community-based suicide prevention program | -evaluation described but no data available |
| *Screening for Depression and Thoughts of Suicide* [60]  Niven (2007) | -Alaska Native  -Norton Sound Region, Alaska | -Identify and support people at risk | -implementation of basic screening form by community health workers (Alaska Native Community Health Aides)  -community-level referral of at-risk patients to behavioral health services providers | -evaluation described but no data available |
| *American Indian Suicide Prevention Model* [61]  Muehlenkamp et al. (2009) | -Northern plains tribes  -North Dakota  -college aged | -Promote connectedness  -Teach coping and problem-solving skills  -Identify and support people at risk | -program workshops and educational seminars  -American Indian medicine wheel “holistic” model of suicide prevention (connections between education, cultural/spiritual, emotional health and physical health support services) | -Suicide knowledge |
| *Applied Suicide Intervention Skills (ASIST)* [62,63]  Hymer et al. (2009)  Cwik et al. (2016) | -Inupiat, Yup’ik; White Mountain Apache  -Norton Sound region, Alaska; Arizona | -Teach coping and problem-solving skills  -Identify and support people at risk | -two-day standardized gatekeeper trainings for community leaders  -suicide first-aid skills and self-care for gatekeepers | -Suicide knowledge  -Self-efficacy to identify and help youth |
| *Nursing Intervention* [64]  Long (2009) | -Wind River Plains  -Wyoming | -Identify and support people at risk  -Lessen Harms and Prevent Future Risk  -Create Protective Environments | -health education provided by community health nurses  -community activity and discussion groups  -therapy for at-risk patients  -coordination with law enforcement to avoid incarceration | -no evaluation data available |
| *Qungasvik (Yup’ik Intervention Toolbox)* [40,65–68]  Allen et al. (2009)  Henry et al. (2012)  Allen et al. (2014)  Rasmus et al. (2014)  Mohatt et al. (2014)  Allen et al. (2018) | -Yup’ik  -Alaska  -Ages 12-18 | -Teach coping and problem-solving skills | -trained community members use Qungasvik toolbox manual to guide community-initiated and customized intervention promoting reasons for life and sobriety  -each module promotes one element Yup’ik cultural engagement or social-emotional learning among youth | -Suicide risk and protective factors |
| *IHS Environmental Health Officer* [69]  Hymer et al. (2010) | -tribes with assigned IHS Environmental Health Officers | -Create protective environments  -Identify and support people at risk | -training, coalition building and partnerships with community resources  -project design, implementation and evaluation  -Applied Suicide Intervention Skills Training Program implementation  -safe firearm storage program | -evaluation described but no data available |
| *IHS Suicide Prevention Initiative* [70]  Dorgan (2010) | -12 area IHS offices | -Identify and support people at risk  -Lessen harms and prevent future risk | -website with youth suicide resources  -suicide assessment and prevention education at substance abuse youth treatment centers  -collaborations with tribal advocacy groups | -no evaluation data available |
| *Our Life* [44]  Goodkind et al. (2012) | -unspecified tribal community  -ages 7-17 | -Promote connectedness  -Teach coping and problem-solving skills | -six-month, trauma-informed, equine-assisted intervention with youth and one family member | -Suicide risk and protective factors |
| *Native American Horse Program* [71]  Spence et al. (2013) | -tribal communities  -Oregon  -youth | -Promote connectedness | -Mustangs and MOHR (Mustangs Offering Hope and Renewal)  -weekly sessions of 2-4 hours each  -gentle contact between youth and horse (horse orientation, horse care, holistic horsemanship, horse riding) | -evaluation described but no data available |
| *Suicide and Self-Injury Surveillance System* [72]  Tingey et al. (2013) | -White Mountain Apache  -Arizona | -Strengthen access and delivery of suicide care  -Identify and support people at risk  -Lessen harms and prevent future risk | -legally mandated suicidal ideation or attempt reporting and referral  -in-person follow-up of all reports by paraprofessional Apache Suicide Prevention Team (ASPT)  -ASPT conducts referrals to available mental health services | -evaluation described but no data available |
| *Mind Body Awareness Project* [73]  Le et al. (2015) | Confederated Salish and Kootenai Tribes  -Montana  -age: 17 | -Teach coping and problem-solving skills -Promote connectedness | -9 module Mind Body Awareness curriculum delivered as a school elective class with homework assignments for each module  -55 minute sessions, 4 per week, over 10 weeks  -trained facilitators from local Confederated Salish and Kootenai Tribes community | -Suicide risk and protective factors  -Suicidal thoughts |
| *Promoting Community Conversations about Research to End Suicide (PC CARES)* [74–76]  Wexler et al. (2015)  Wexler et al. (2016)  Wexler et al. (2017) | -Alaska Native  -Alaska | -Promote Connectedness  -Identify and Support People at Risk | -share recent suicide prevention research with village providers at monthly community learning circles  -create community of providers who monthly discuss “what we know, think, and want to do” to prevent suicide | -evaluation described but no data available |
| *Suicide Prevention Program* [51]  Kelley et al. (2015) | -tribal community  -Montana | -Create protective environments  -Promote connectedness  -Identify and support people at risk | -community-driven approach to suicide prevention  -increase understanding of suicide prevention  -help individuals in need through culturally based coordination of services  -promote community involvement partnerships | -Conducted formative, qualitative research to better understand suicide prevention in the community |
| *Celebrating Life surveillance system* [63]  Cwik et al. (2016) | -White Mountain Apache Tribe  -Arizona | -Teach coping and problem-solving skills  -Identify and support people at risk | -community surveillance system to track suicide deaths, attempts, and ideation  -formal treatment services for identified at-risk individuals, including referrals for mental health care  -public education campaign, including posters, public service announcements, and talk shows  -door-to-door educational campaign about the surveillance system, services, and national suicide lifeline  -gatekeeper training for community health and emergency assistance providers  -Sources of Strength suicide prevention program in schools | -Deaths by suicide |
| *Kognito Gatekeeper Simulations (KGS)* [37]  Bartgis et al. (2016) | -100% of participants self-identified as AIAN (data were a subset of a larger study) | -Identify and support people at risk | -online role play gatekeeper training delivered to teachers  -mini-conversation games where users interact with animated multiethnic student avatars experiencing psychological distress  -virtual role-plays are used to facilitate motivational interviewing | -Suicide protective factors  -Suicidal ideation |
| *Lumbee Rite of Passage* [77]  Langdon et al. (2016) | -Lumbee -North Carolina  -ages 11-19 | -Promote connectedness  -Teach coping and problem-solving skills | -curriculum to educate youth about risk factors for injury and strategies to address those factors  -community forum and youth panel focused on youth coping strategies for bullying and stress | -no evaluation data available |
| *New Hope* [39]  Cwik, et al. (2016) | -White Mountain Apache  -Arizona  -ages 10-19 | -Identify and support people at risk | -local community mental health workers deliver brief intervention and follow-up to adolescents with recent suicide attempt | -Suicide risk factors |
| *Village Wellness Team Program* [78]  de Schweinitz et al. (2017) | -Alaska Native; Western Athabascan  -Alaska | -Create Protective Environments  -Promote Connectedness  -Identify and Support People at Risk | -dedicated tribal wellness volunteer group  -community response team to attend to individuals with active suicidal ideation and intent  -distribution of gun safes | -Qualitative research exploring program impacts and recommendations for wellness and prevention |
| **Child Abuse/Neglect (n =3 implementations)** | | | | |
| *Child Abuse Prevention Coordinator* [56]  DeBruyn et al. (1997) | -New Mexico | -Intervene to lessen harms and prevent future risk | - dedicated tribal health/social services coordinator to help connect families to the preventive services they need to stop violence against children before it occurs  -qualitative data exploring program impacts and recommendations for wellness and prevention | -no evaluation data available |
| *SafeCare* [38]  Chaffin et al. (2012) | -tribal communities  -Oklahoma  -age 29 | -Enhance parenting skills to promote healthy child development  -Intervene to lessen harms and prevent future risk | -home-based visitor program  -manualized, highly structured behavioral skill training | -Child abuse and neglect risk factors  -Child abuse and neglect recidivism |
| *Pathway to Hope (PTH)*  [43]  Payne et al. (2013) | -Alaska Native  -Alaska | - Enhance parenting skills to promote healthy child development | -PTH video, guidebook, and 3 day community facilitator training  -education on child sexual abuse, indigenous healing and wellness, and community empowerment strategies  -strengths-based solutions curriculum delivered to community leaders | -evaluation described but no data available |
| **Youth Violence (n = 4 implementations)** | | | | |
| *Families and Schools Together (FAST)* [46]  Kratochwill et al. (2004) | -three tribes  -Wisconsin  -ages 4-9 | -Promote Family Environments that Support Healthy Development | -activity-based collaborative program to increase protective factors for at-risk children  -structured curriculum with unstructured play and socialization time  -initial home visit, then 8 weekly meetings then monthly family meetings | -Youth violence risk factors |
| *Tribal Youth Program* [79]  Hurst et al. (2006) | -available to all tribal groups | -Intervene to Lessen Harms and Prevent Future Risk  -Connect Youth to Caring Adults and Activities | -coordination of community infrastructure to control crime, violence, and drug abuse  -development of youth tribal courts that incorporate cultural traditions  -youth mentoring programs with police officers  -home detention systems instead of youth incarceration | -no evaluation data available |
| *Bully-Proofing Your School* [80]  Gallagher et al. (2008) | -Lac Vieux Desert Band of Lake Superior Chippewa Indians  -Michigan | -Connect Youth to Caring Adults and Activities  -Create Protective Community Environments | -system-wide universal prevention program with “comprehensive school climate change”  -bullying/violence awareness training for all school staff  -bullying avoidance and victimization resistance training provided to all classroom groups | -Bullying behaviors |
| *Gang Reduction through Intervention, Prevention, and Suppression (GRIPS)* [81]  Trottier (2009) | -Aberdeen Area, South Dakota | -Strengthen Youth’s Skills  -Connect Youth to Caring Adults and Activities  -Create Protective Community Environments | -local tribal gang ordinance to empower school discipline, housing authority safety, and judicial discipline  -preemptive parental notification requests to involve parents  -judicially mandated Young Warriors Program two-day intervention seminar for youth identified in gang-related activities  -graffiti abatement program  -youth basketball tournament with violence prevention public service announcements  -gang prevention training provided to community members, school staff and primary, middle and high school students | -Youth violence risk factors  -Youth violence behaviors  -Tribal court actions |
| **Intimate Partner Violence and Sexual Violence (n = 4 implementations)** | | | | |
| *Alaska: Program reduces rapes by 30 percent* (no program name available) [82]  Crime Control Digest (2000) | -Alaska Natives  -Anchorage, Alaska | -Engage influential adults and peers  -Create protective environments | -police partnerships with Alaska Native community to provide escorts and safety guidelines to new residents  -brochures and posters in bars  -bartender education on spotting sexual violence | -Reported rapes |
| *Domestic Violence Pilot Project* [83]  Cullen et al. (2003) | -Rosebud IHS Health Center (South Dakota)  -Choctaw Nation Health Center (Oklahoma) | -Support survivors to increase safety and lessen harms | -based in Indian Health Service health care facilities and community-based health care delivery  -routine screening of patients for domestic violence and intimate partner violence  -Proper training for doctors, nurses and allied health providers on how to screen for abuse | -no evaluation data available |
| *Promoting Healthy Relationships Project* [45]  Richmond et al. (2008) | -two Arizona tribal nations  -Arizona  -11th and 12th grade students | -Teach safe and healthy relationship skills  -Teach skills to prevent sexual violence  -Engage influential adults and peers | -10 or 11 session dating violence prevention curriculum taught in the classroom  -youth development programing outside of school (i.e. sports, recreation, community fairs) | -no evaluation data reported |
| *Discovery Dating* [84]  Schanen et al. (2017) | -Western United States tribal middle schools  -7th grade students | -Teach safe and healthy relationship skills  -Teach skills to prevent sexual violence | -core curriculum including goal setting, examination of benefits/pitfalls of relationships, impacts of decision making, reliance on facts and information, mentoring, management of chaos  -strength-based classroom activities around goal setting, envisioning a better future, and cultural affirmation | -Intimate partner and sexual violence risk factors  Intimate partner and sexual violence protective factors |
| **Elder Abuse (n = 1 implementation)** | | | | |
| *Family Care Conference* [85]  Holkup (2003) | -High Plains nation  -Montana | -Teach Skills to Prevent Elder Abuse | -elder-focused, family-centered, community-based intervention  -family group conference  -referral, screening, logistical preparations, family meeting, and follow-up | -evaluation described but no data available |
| **Cross-cutting Violence Areas (more than one form of violence identified for prevention) (n = 6 implementations)** | | | | |
| *Special Initiatives Team* [86]  DeBruyn et al. (1988) | -adaptable to any tribal community | -Identify and support people at risk  -Teach coping and problem-solving skills | -national crisis response team from Indian Health Service  -crisis and prevention consultation in response to violent behaviors  -program planning, development, and evaluation; resource mobilization; data collection and surveillance; local needs analysis | -evaluation described but no data available |
| *Safe Futures Initiative* [87]  Guilmet et al. (1998) | -Puyallup Indian Reservation  -Washington | -Strengthen Youth’s Skills  -Connect Youth to Caring Adults and Activities  -Identify and support people at risk  -Teach coping and problem-solving skills  -Enhance parenting skills to promote healthy child development | -Safe Futures Initiative built on two existing programs: Positive Reinforcement in Drug Education (PRIDE) and Puyallups Against Violence (PAV)  -teachers trained to implement social learning strategies  -improved student peer supports and counseling staff  -parenting skills training  -dropout retrieval/truancy prevention program | -evaluation described but no data available |
| *Alcohol policy and availability of police services* [36]  Wood et al. (2006) | -132 Alaska Native villages  -Alaska | -Create protective environments | -alcohol policies to limit the sale, importation and/or possession of alcohol within tribal villages  -presence of local police authority (local police, village public safety officer and/or Alaska state trooper) | -Alcohol policy  -Police presence  -Nonfatal injuries |
| *Family Wellness Warriors Initiative (FWWI)* [88]  Gottlieb et al. (2011) | -Alaska Native  -Alaska | -Promote Connectedness  -Teach coping and problem-solving skills  -Create Protective Community Environments  -Engage influential adults and peers | -FWWI provides program structure, core philosophy and framework; tribes plan implementation  -variety of trainings offered (1 day or 1-2 week sessions)  -courses reinforce the power of empathy, compassion, and relationship building  -transitional living training for incarcerated participants  -faith-based, cultural component with monthly support groups and quarterly follow-up meetings | -Violence protective factors  -Violence behaviors (criminal justice system involvement) |
| *Values-Based Career Intervention* [89]  Garcia (2012) | -Salt River Pima-Maricopa  -Arizona  -92% Pima-Maricopa; 8% Tohono O’odham Nation  -Age: 16.31 | -Strengthen Youth’s Skills | -modeled after Talented At-Risk Girls: Encouragement and Training for Sophomores (TARGETS)  -activities to facilitate life-planning and goal-setting  -5 hour session in university counseling department clinic  -group lessons and exercises followed by one-on-one career counseling | -Violent behaviors (suicidality, gang behavior)  -Violence protective factor |
| *Arrowhead Business Group- Apache Youth Entrepreneurship Program (ABG)* [90]  Tingey et al. (2016) | -White Mountain Apache  -Arizona  -ages 13-16 | -Strengthen Youth’s Skills  -Connect Youth to Caring Adults and Activities  -Teach coping and problem-solving skills | -16 lesson curriculum (60 hours over 8 months) focusing on entrepreneurship, business development, life skills, self-efficacy, and finance  -taught in residential summer camp and school workshops | -evaluation described but no data available |

^1^ When a program entry includes multiple authors, this represents multiple study publications describing the same program. Sometimes, these publications represent different stages of program development, implementation, and evaluation. At other times, they report data from iterations of the program implemented in different tribal populations or in different years.

^2^ If not reported, all study participants identify as American Indian or Alaska Native. If not reported, other demographic information (e.g., age, location) were unavailable. Whenever possible, tribe was reported.

^3^ Centers for Disease Control and Prevention. Technical Packages for Violence Prevention: Using evidence-based strategies in your violence prevention efforts. National Center for Injury Prevention and Control. Updated November 30, 2020. Accessed December 2, 2021. https://www.cdc.gov/violenceprevention/communicationresources/pub/technical-packages.html

^4^ Study methodology varied and the following were represented: pre-post test design, intervention/control group (non-randomized), intervention/control group (randomized), no control group, passive or active surveillance, surveys or questionnaire self-report, qualitative and/or quantitative interviews, focus groups. At times, information was just mentioned with no further details.
